# Direct Medical Costs of Tetanus, Dengue, and Sepsis Patients in an Intensive Care Unit in Vietnam

**DOI:** 10.3389/fpubh.2022.893200

**Published:** 2022-06-20

**Authors:** Trinh Manh Hung, Nguyen Van Hao, Lam Minh Yen, Angela McBride, Vu Quoc Dat, H. Rogier van Doorn, Huynh Thi Loan, Nguyen Thanh Phong, Martin J. Llewelyn, Behzad Nadjm, Sophie Yacoub, C. Louise Thwaites, Sayem Ahmed, Nguyen Van Vinh Chau, Hugo C. Turner, The Vietnam ICU Translational Applications Laboratory (VITAL) Investigators, Dang Phuong Thao

**Affiliations:** ^1^Oxford University Clinical Research Unit, Wellcome Trust Major Overseas Programme, Ho Chi Minh City, Vietnam; ^2^Hospital for Tropical Diseases, Ho Chi Minh City, Vietnam; ^3^Department of Infectious Diseases, University of Medicine and Pharmacy, Ho Chi Minh City, Vietnam; ^4^Department of Global Health and Infection, Brighton and Sussex Medical School, Brighton, United Kingdom; ^5^Department of Infectious Diseases, Hanoi Medical University, Hanoi, Vietnam; ^6^Oxford University Clinical Research Unit, Wellcome Trust Major Overseas Programme, Hanoi, Vietnam; ^7^Centre for Tropical Medicine and Global Health, Nuffield Department of Medicine, University of Oxford, Oxford, United Kingdom; ^8^Medical Research Council (MRC) Unit the Gambia at the London School of Hygiene & Tropical Medicine, Fajara, Gambia; ^9^MRC Centre for Global Infectious Disease Analysis, School of Public Health, Imperial College London, Norfolk Place, London, United Kingdom

**Keywords:** dengue, sepsis, tetanus, direct medical cost, ICU, Vietnam

## Abstract

**Background:**

Critically ill patients often require complex clinical care by highly trained staff within a specialized intensive care unit (ICU) with advanced equipment. There are currently limited data on the costs of critical care in low-and middle-income countries (LMICs). This study aims to investigate the direct-medical costs of key infectious disease (tetanus, sepsis, and dengue) patients admitted to ICU in a hospital in Ho Chi Minh City (HCMC), Vietnam, and explores how the costs and cost drivers can vary between the different diseases.

**Methods:**

We calculated the direct medical costs for patients requiring critical care for tetanus, dengue and sepsis. Costing data (stratified into different cost categories) were extracted from the bills of patients hospitalized to the adult ICU with a dengue, sepsis and tetanus diagnosis that were enrolled in three studies conducted at the Hospital for Tropical Diseases in HCMC from January 2017 to December 2019. The costs were considered from the health sector perspective. The total sample size in this study was 342 patients.

**Results:**

ICU care was associated with significant direct medical costs. For patients that did not require mechanical ventilation, the median total ICU cost per patient varied between US$64.40 and US$675 for the different diseases. The costs were higher for patients that required mechanical ventilation, with the median total ICU cost per patient for the different diseases varying between US$2,590 and US$4,250. The main cost drivers varied according to disease and associated severity.

**Conclusion:**

This study demonstrates the notable cost of ICU care in Vietnam and in similar LMIC settings. Future studies are needed to further evaluate the costs and economic burden incurred by ICU patients. The data also highlight the importance of evaluating novel critical care interventions that could reduce the costs of ICU care.

## Background

Critically ill patients require complex clinical care by highly trained staff, often within a specialized intensive care unit (ICU) with advanced equipment. The provision of high-quality critical care in low-and middle-income countries (LMICs) is challenging due to the burden these requirements place on infrastructure, healthcare expenditure and human resources (1–3). Furthermore, the limited data available indicate that the number of critical care beds in LMICs are significantly lower than high-income countries (4–8).

The requirement for high numbers of specialized staff and advanced medical equipment means that critical care is one of the most expensive types of hospital-based care globally. In the United States (US), ICU costs have been estimated to account for around 13 to 39% of the total hospital costs nationally (9, 10). The daily ICU costs in high-income countries that have been reported vary from US$1,464 to US$4,518 (adjusted to 2019 prices) (11–13). Whilst critical care is less expensive in LMICs (partly because of lower labor costs), cost data are limited (8). The daily ICU costs that have been reported in Thailand, Malaysia, China and India vary from US$292 to US$1,421 (adjusted to 2019 prices) (14–17). These costs are equivalent to 2.2 to 4.0 times the countries 2018 per capita health care expenditure (18). This highlights the significance of ICU costs in LMICs and the need for further data.

Similar to other LMICs, the healthcare system in Vietnam has limited human resources, infrastructure and technology to provide quality health care and critical care (19). A recent study reported that the number of ICU beds only accounted for approximately 2.5% of the total hospital beds of the surveyed hospitals (20). Patients with infectious diseases remain a significant population in LMIC ICUs, including Vietnam. Within the Hospital for Tropical Diseases in Ho Chi Minh City (HCMC) (a tertiary referral hospital specialized in infectious diseases serving the south of Vietnam) tetanus, sepsis, and dengue accounted for roughly 81% of total patients admitted to ICU annually (19). Further epidemiological information on these diseases is provided in Supplementary Box 1.

Improving knowledge of costs of critical care is important for evaluating the cost-effectiveness of new interventions and for better resource utilization. However, there is currently limited information available related to the health care cost of hospitalized patients who need critical care, particularly for those with infectious diseases. particularly the out-of-pocket costs. Although, social health insurance has been shown to decrease the out-of-pocket costs of patients (21, 22), they can still be notable (23, 24). These out-of-pocket costs are particularly important to investigate in the context of critical care, due to its potentially high costs; which may mean they remain significant even for patients that are covered by insurance. By the end of 2019, the coverage of the national health insurance program was almost 90% of the total Vietnamese population. Despite this, around this time, catastrophic health expenditure (when defined as occurring when the patient's out-of-pocket health care payment was >10% of the average annual household expenditure in Vietnam), was estimated to incur in 47% of patients with septic shock and 13% of those with dengue shock admitted to the Hospital for Tropical Diseases ICU. In fatal cases, these were 56 and 84% respectively (25).

Poorer groups of the population will be most at risk of the negative consequences of these out-of-pocket costs generating socio-economic difficulties and exacerbating equity and accessibility issues (24, 26).

Limited information related to the cost of critical care illness, particularly for infectious diseases, has been reported in Vietnam. It is important to understand the potential significance of these costs and the degree the costs may vary according to disease and associated severity. This study aims to investigate the direct medical cost (defined as costs directly related to medical services) in the context of critical care for key infectious diseases (tetanus, sepsis, and dengue) in a referral hospital in HCMC, Vietnam, explore how the costs and cost drivers can vary and investigate the patients' out-of-pocket costs in the context of the national health insurance program. We focus on these key diseases due to their notable burden in this setting and the availability of data for them.

## Method

We calculated the direct medical costs for patients requiring critical care for tetanus, dengue and sepsis. Costing data, extracted from the bills of patients hospitalized to adult ICU in the Hospital for Tropical Diseases in HCMC from January 2017 to December 2019 were retrieved from three different studies of tetanus, dengue and sepsis that have been conducted in the hospital (details of the data collection procedure and the primary studies are shown in Supplementary Figure 1) (27–29). The costs were considered from the health sector perspective which only includes the costs associated with the health sector, such as the costs covered by the health insurance program and the patient's copayment for the medical services. It was not possible to involve patients or the public in the design, conduct and reporting of this study.

The Hospital for Tropical Diseases was chosen because it is the only referral hospital dedicated to the treatment of infectious diseases in HCMC and serves patients from a wide geographical area across the south of Vietnam.

Treatment for all the specified diseases followed national Vietnamese guidelines (30, 31). In brief patients were treated with antibiotics routinely or if clinically indicated in dengue and sepsis. Patients with organ failure were provided with appropriate organ support, such as mechanical ventilation, inotropes or continuous renal replacement therapy. Nutrition was provided enterally in those unable to swallow and intravenous fluids given as clinically indicated. Muscle paralysis was reserved for those with ventilator asynchrony or acute respiratory distress syndrome, except those with tetanus, in whom it was routinely administered to those with severe spasms interfering with respiration.

### Data Collection

For the three studies mentioned above, we collected and entered into a database the patients' bill for estimating the costs directly related to medical services received during the patient's ICU admission. In total, we extracted the information from 342 patients. The costs incurred during their ICU stay were grouped into defined cost categories (Table 1). The costs related to antibiotics were extracted to examine the contribution of this cost to the total medication costs. The total cost of the patients' hospital admission was also extracted from the bills, alongside certain background information (such as gender, year of birth, home province, health insurance status, admission and discharge date). Where available on the bill, the amount reimbursed by the national health insurance program was extracted. Mortality data were extracted from the case report forms. In Vietnam, cultural practices are such that the very severely ill are often discharged home at the request of their families for end-of-life care when recovery is no longer considered likely. Such patients were assumed to have died at home.

**Table 1:** Definition of cost categories.

**Type of cost**	**Detail**
Laboratory costs	Costs related to laboratory tests and imaging
ICU bed charges	Daily ICU bed charges
Medication costs	Costs related to fluids and medications
Procedure costs	Costs associated with procedures, such as mechanical ventilation, hemofiltration and other essential techniques required during ICU stay
Medical nutrition costs (Dietary cost)	Costs related to nutrition that was prescribed by physicians during the ICU stay
Consumable costs	Costs related to consumable items during ICU stay
Other costs	Costs that did not fall within the above groups

### Cost Estimates

Based on the data from the hospital bills, we reported both the direct medical cost associated with the patients' ICU care and their total hospital admission (the latter also includes costs incurred from care in other wards). We also reported the ICU costs stratified by key cost categories. Moreover, the daily cost of ICU was calculated by dividing the total direct medical costs incurred in ICU by the length of stay in ICU. The costs were stratified based on whether the patient needed mechanical ventilation, as a proxy for the severity of the cases (32). As there is no overall indicator of disease severity validated for all three diseases, mechanical ventilation was chosen as a marker of severity as it was clearly indicative of single organ system failure and patients requiring mechanical ventilation require increased input in staff time, equipment and drugs. This measure was also chosen to make the results comparable to other settings, for example those with high dependency units, where generally only patients requiring mechanical ventilation are admitted to ICU. It should be noted (and is discussed further in the limitations section) that when stratifying by mechanical ventilation status, our sample sizes for certain subgroups for dengue and sepsis cases were small (Table 2).

**Table 2:** Overview of characteristics of the patients.

	**Dengue**		**Sepsis**		**Tetanus**	
	**No mechanical ventilation [*N* = 15]**	**Received mechanical ventilation** **[*N* = 13]**	**No mechanical ventilation [*N* = 6]**	**Received mechanical ventilation [*N* = 36]**	**No mechanical ventilation [*N* = 141]**	**Received mechanical ventilation [*N* = 131]**
**Gender** ***n*** **(%)**						
Female	9 (60.0)	8 (61.5)	3 (50.0)	9 (25.0)	20 (14.2)	23 (17.6)
Male	6 (40.0)	5 (38.5)	3 (50.0)	27 (75.0)	121 (85.8)	108 (82.4)
**Address** ***n*** **(%)**					
Ho Chi Minh	8 (53.3)	4 (30.8)	4 (66.7)	11 (30.6)	28 (19.9)	25 (19.1)
Other provinces	7 (46.7)	9 (69.2)	2 (33.3)	25 (69.4)	113 (80.1)	106 (80.9)
**Age**						
Mean (SD)	30.7 (13.7)	33.9 (14.8)	57.3 (11.4)	51.9 (17.0)	46.4 (13.6)	51.9 (14.8)
Median (Q1, Q3)	30.0 (19.0, 38.0)	29.0 (21.0, 41.0)	59.5 (51.5, 66.0)	50.5 (42.0, 61.0)	45.0 (38.0, 56.0)	53.0 (42.0, 61.0)
**Covered by the national health insurance program** ***n*** **(%)**						
No	8 (53.3)	1 (7.7)	0 (0)	12 (33.3)	0(0)	0(0)
Yes	7 (46.7)	12 (92.3)	6 (100)	24 (66.7)	0 (0)	0 (0)
Not available	0(0)	0(0)	0(0)	0(0)	141 (100)	131 (100)
**Duration of hospital admission (days)**						
Mean (SD)	5.1 (1.58)	12.1 (8.10)	15.0 (5.18)	18.3 (15.0)	18.7 (5.33)	34.5 (20.2)
Median (Q1, Q3)	5.0 (4.0, 6.0)	14.0 (4.0, 15.0)	14.0 (12.3, 16.5)	16.0 (6.0, 25.0)	19.0 (14.0, 22.0)	30.0 (26.0, 37.0)
**Days in ICU**						
Mean (SD)	2.0 (1.1)	10.6 (5.7)	6.3 (4.5)	14.3 (11.6)	9.4 (4.1)	26.6 (19.3)
Median (Q1, Q3)	2.0 (1.0, 2.5)	10.0 (5.00, 16.0)	5.0 (3.5, 6.5)	11.5 (6.0, 16.3)	9.0 (6.0, 12.0)	23.0 (19.0, 28.0)
**Mortality** ***n*** **(%)**						
No	15 (100)	6 (46.2)	5 (83.3)	19 (52.8)	140 (99.3)	125 (95.4)
Yes	0 (0)	7 (53.8)	1 (16.7)	17 (47.2)	1 (0.7)	6 (4.6)

*Q1: 25% quartile and Q3: 75% quartile*.

Due to the highly skewed data, we reported the median and interquartile range for the costs and cost categories. For patients covered by the national health insurance program, we estimated the proportion of the total ICU cost that was covered by the program and the remaining cost that would be covered by the patient (out-of-pocket cost). This was based on information from the patients' bill which included the total ICU cost and the amount reimbursed by the health insurance program. It should be noted that only insurance information related to the national health insurance program was available for this study and the potential contribution from private medical insurance was not considered.

The costs reported in different years were adjusted for inflation using Vietnam Gross Domestic Product deflator rates (33) and then converted to US Dollars using the average 2019 exchange rate reported by the World Bank (US$1 = 23,050 Vietnamese dong (VND)) (34). All costs are presented in 2019 US dollar prices.

### Ethics Approval

The primary studies from which this sample was drawn were approved by the Oxford Tropical Research Ethics Committee and Hospital for Tropical Diseases Ethics Committee and patients gave written informed consent for their medical bills to be obtained.

## Results

### Baseline Information

The collected baseline information is summarized in Table 2. Among the sample, 270 (78.9%) patients were male, and 262 (76.6%) came from outside HCMC. The median age of patients was 48 (IQR 38, 59) years old. Patients spent a median of 13 days in ICU and 22 days in hospital (Supplementary Table 1). The patients' time in ICU accounted for an average of 45.7 and 75.0% of their time in hospital for non-ventilated and ventilated patients, respectively. There were 28 patients with dengue, 42 patients with sepsis and 272 patients with tetanus. Median ICU length of stay was 3.5, 10, and 15 days for dengue, sepsis and tetanus, respectively.

### The Total Direct Medical Cost of ICU Patients

The costs across the different diseases and severity status are summarized in Table 3. ICU costs accounted for 86.5–91.2% of the patients' total hospital admission costs. The ICU cost of patients that did not require mechanical ventilation was consistently and notably lower than the ICU cost of those requiring mechanical ventilation across the diseases. For the patients that did not require mechanical ventilation, the total ICU cost for patients with sepsis was most expensive. Specifically, the median total ICU cost was US$675 (Interquartile Range (IQR) US$550, US$990), US$273 (IQR US$168, US$503) and US$64.4 (IQR US$55.3, US$95.9) for patients with sepsis, tetanus and dengue, respectively (Table 3). For the patients who required mechanical ventilation, the total ICU cost of those with dengue was the most expensive with a median of US$4,250 (IQR US$2,030, US$6,290), followed by sepsis and tetanus with a median of US$2,860 (IQR US$1,300, US$4,500) and US$2,590 (IQR US$1,900, US$3,230), respectively.

**Table 3:** The median costs for the ICU patients stratified by the different cost categories.

	**Dengue**		**Sepsis**		**Tetanus**	
**Median cost** **(US$)**	**No mechanical ventilation [*N* = 15]**	**Received mechanical ventilation [*N* = 13]**	**No mechanical ventilation [*N* = 6]**	**Received mechanical ventilation [*N* = 36]**	**No mechanical ventilation [*N* = 141]**	**Received mechanical ventilation [*N* = 131]**
ICU bed charges (Q1, Q3)	21.9 (17.8; 40.4)	264 (128; 388)	121 (92.7; 197)	249 (164; 400)	128 (85.8; 180)	592 (471; 716)
Laboratory costs (Q1, Q3)	36.8 (29.5; 49.5)	513 (455; 850)	181 (144; 234)	452 (326; 761)	21.9 (15.5; 33.6)	203 (145; 326)
Procedure costs (Q1, Q3)	0 (0; 0)	1,240 (729; 1,750)	112 (75.3; 112)	545 (280; 1,080)	0 (0; 7.30)	605 (471; 802)
Medication costs (Q1, Q3)	2.88 (2.1; 17.7)	2,150 (574; 2,960)	241 (187; 481)	1,140 (426; 2,020)	112 (31.1; 216)	783 (471; 1,060)
Medical nutrition costs (Q1, Q3)	0 (0; 0)	24.4 (5.0; 66.9)	0 (0; 0)	51.4 (21.6; 95.0)	0 (0; 15.9)	189 (141; 232)
Consumable costs (Q1, Q3)	1.66 (0.9; 2.1)	264 (52.0; 735)	25.2 (17.7; 37.0)	47.2 (22.9; 242)	10.8 (5.2; 18.7)	65.3 (39.1; 91.7)
Other costs (Q1, Q3)	1.82 (1.2; 2.7)	3.42 (0.7; 10.9)	7.74 (5.2; 13.4)	6.26 (2.15; 15.1)	0 (0; 0)	9.29 (3.6; 19.3)
Total ICU cost (Q1, Q3)	64.4 (55.3; 95.9)	4,250 (2,030; 6,290)	675 (550; 990)	2,860 (1,300; 4,500)	273 (168; 503)	2,590 (1,900; 3,230)
Total hospital admission cost (Q1, Q3)	150 (111; 175)	4,250 (2,030; 6,300)	1,340 (1,060; 1,470)	3,130 (1,640; 4,990)	403 (254; 615)	2,710 (1,980; 3,410)

*Costs are in US$ 2019 prices. Q1: 25% quartile and Q3: 75% quartile*.

**Figure 1:**
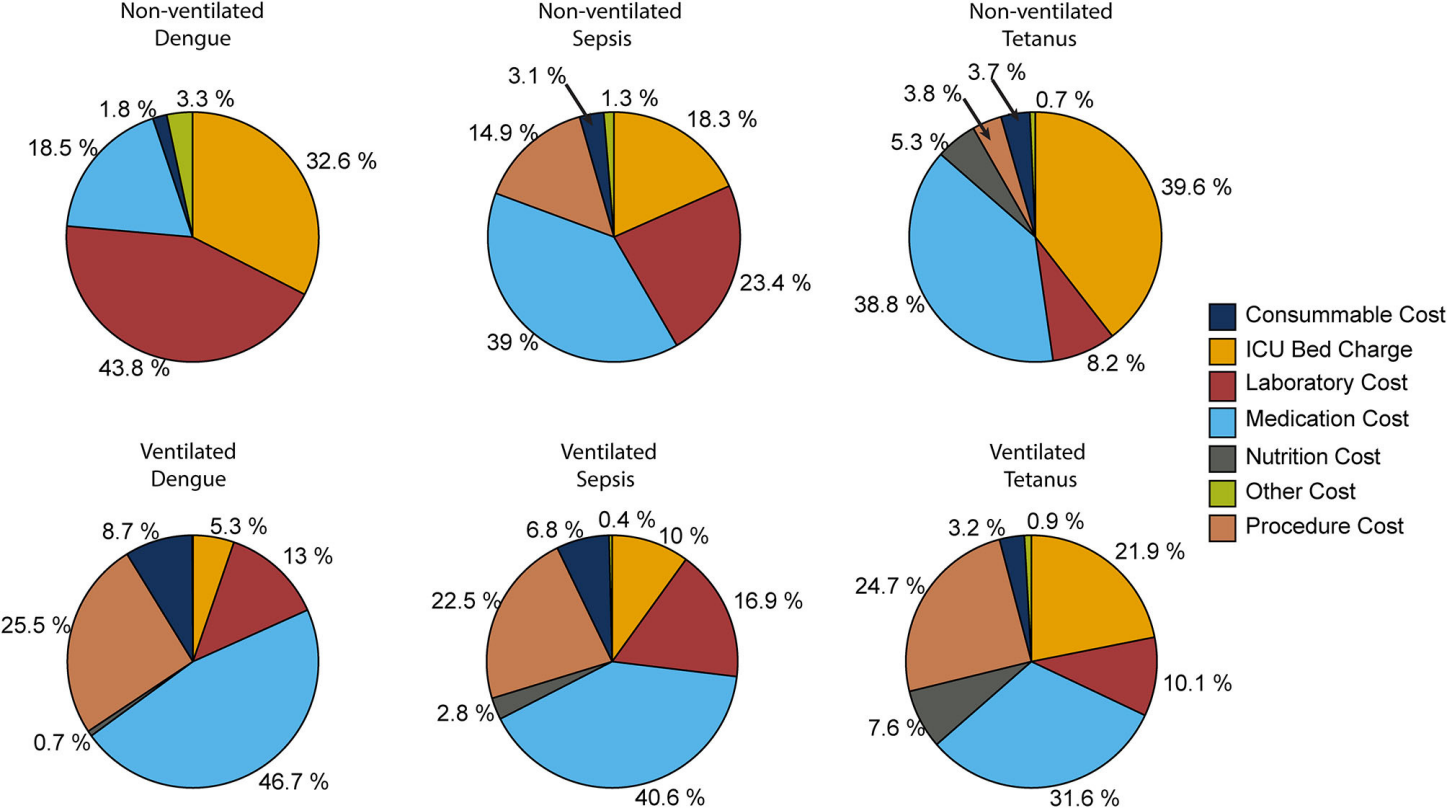
Percentage share of the different ICU cost categories.

The key drivers of ICU costs also differed across the diseases and the severity of the cases (Figure 1). For patients with dengue who did not require mechanical ventilation, the two most expensive cost categories were laboratory costs and ICU bed charges. For sepsis, these were medication costs and laboratory costs, and for tetanus, they were medication costs, and the ICU bed charges. In contrast, in patients requiring mechanical ventilation, the two most expensive cost categories for all three of the diseases were the medication costs and procedure costs. Interestingly, for mechanical ventilated tetanus patients, the ICU bed charges were also a major cost driver (Figure 1), likely due to their duration of stay in ICU (Table 2).

### Daily Costs of ICU Patients

The total direct medical cost is highly influenced by the duration of ICU stay. We therefore also calculated the average daily ICU costs across the different diseases. Table 4 describes the median daily cost of patients during their ICU stay across the different diseases and severity status for the different cost categories.

**Table 4:** The median daily costs for the ICU patients stratified by the different cost categories.

	**Dengue**		**Sepsis**		**Tetanus**	
**Median ICU cost (US$) per day**	**No mechanical ventilation [*N* = 15]**	**Received mechanical ventilation [*N* = 13]**	**No mechanical ventilation [*N* = 6]**	**Received mechanical ventilation [*N* = 36]**	**No mechanical ventilation [*N* = 141]**	**Received mechanical ventilation [*N* = 131]**
ICU bed charges (Q1, Q3)	14.6 (14.6; 17.8)	25.1 (24.7; 26.4)	30.0 (28.5; 30.6)	25.7 (23.1; 27.4)	14.6 (14.3; 15.0)	26.2 (24.9; 26.9)
Laboratory test costs (Q1, Q3)	21.6 (16.8; 30.0)	65.3 (39.5; 94.3)	41.4 (31.9; 47.9)	48.6 (29.6; 61.9)	2.5 (1.9; 3.7)	9.3 (7.1; 12.0)
Procedure costs (Q1, Q3)	0 (0; 0)	98.3 (61.0; 223)	22.4 (12.3; 28.7)	45.5 (30.2; 85.1)	0 (0; 0.6)	27.6 (23.8; 31.0)
Medication costs (Q1, Q3)	2.2 (1.5; 9.1)	159 (87.1; 537)	60.8 (56.2; 67.4)	90.7 (54.9; 142)	13.0 (5.7; 18.8)	33.1 (24.0; 43.2)
Medical nutrition costs (Q1, Q3)	0 (0; 0)	2.2 (0.5; 4.9)	0 (0; 0)	4.9 (3.1; 6.8)	0 (0; 1.9)	8.2 (7.4; 9.0)
Other costs (Q1, Q3)	1.2 (0.8; 1.9)	0.2 (0.1; 0.8)	1.70 (1.1; 2.3)	0.6 (0.3; 1.3)	0 (0; 0)	0.4 (0.2; 0.8)
Total daily ICU cost (Q1, Q3)	47.5 (38.0; 57.2)	406 (202; 973)	152 (145; 160)	213 (160; 341)	33.3 (25.2; 42.1)	109 (93.2; 124)

*Costs are in US$ 2019 prices. Q1: 25% quartile and Q3: 75% quartile*.

For patients not requiring mechanical ventilation, the daily cost of sepsis was the most expensive (median of US$152 (IQR US$145, US$160)), followed by dengue (median of US$47.5 (IQR US$38.0, US$57.2)) and tetanus (median of US$33.3 (IQR US$25.2, US$42.1)). For the patients requiring mechanical ventilation, the median daily cost of dengue was the most expensive with a median of US$406 (IQR US$202, US$973), followed by sepsis with a median of US$213 (IQR US$160, US$341) and tetanus with a median of US$109 (IQR US$93.2, US$124). As with the total cost, the key drivers for the daily costs differed across the diseases and the severity of the cases. For the patients not requiring mechanical ventilation, the most expensive cost categories were ICU bed charges for patients with tetanus, laboratory costs for those with dengue and medication costs for patients with sepsis. In contrast, for patients requiring mechanical ventilation, costs related to medication were consistently the most expensive daily cost category (Table 4). The difference between the calculated daily ICU costs for the three diseases was wider than the difference between their calculated total ICU costs (Tables 3, 4).

### The Usage and Cost of Antibiotics in ICU

For the patients who did not require mechanical ventilation, the usage and cost of antibiotics in the ICU was relatively limited in patients with dengue and tetanus but as expected, for patients with sepsis was considerably higher with a median of US$172 (IQR US$155, US$233) (Supplementary Table 2). In contrast, all patients requiring mechanical ventilation had substantial antibiotic costs. Specifically, the median cost of antibiotic use in sepsis, dengue and tetanus were US$415 (IQR US$160, US$783), US$267 (IQR US$146, US$616) and US$75 (IQR US$0.3, US$329), respectively (Supplementary Table 2).

### Contribution From the National Health Insurance Program

**Figure 2:**
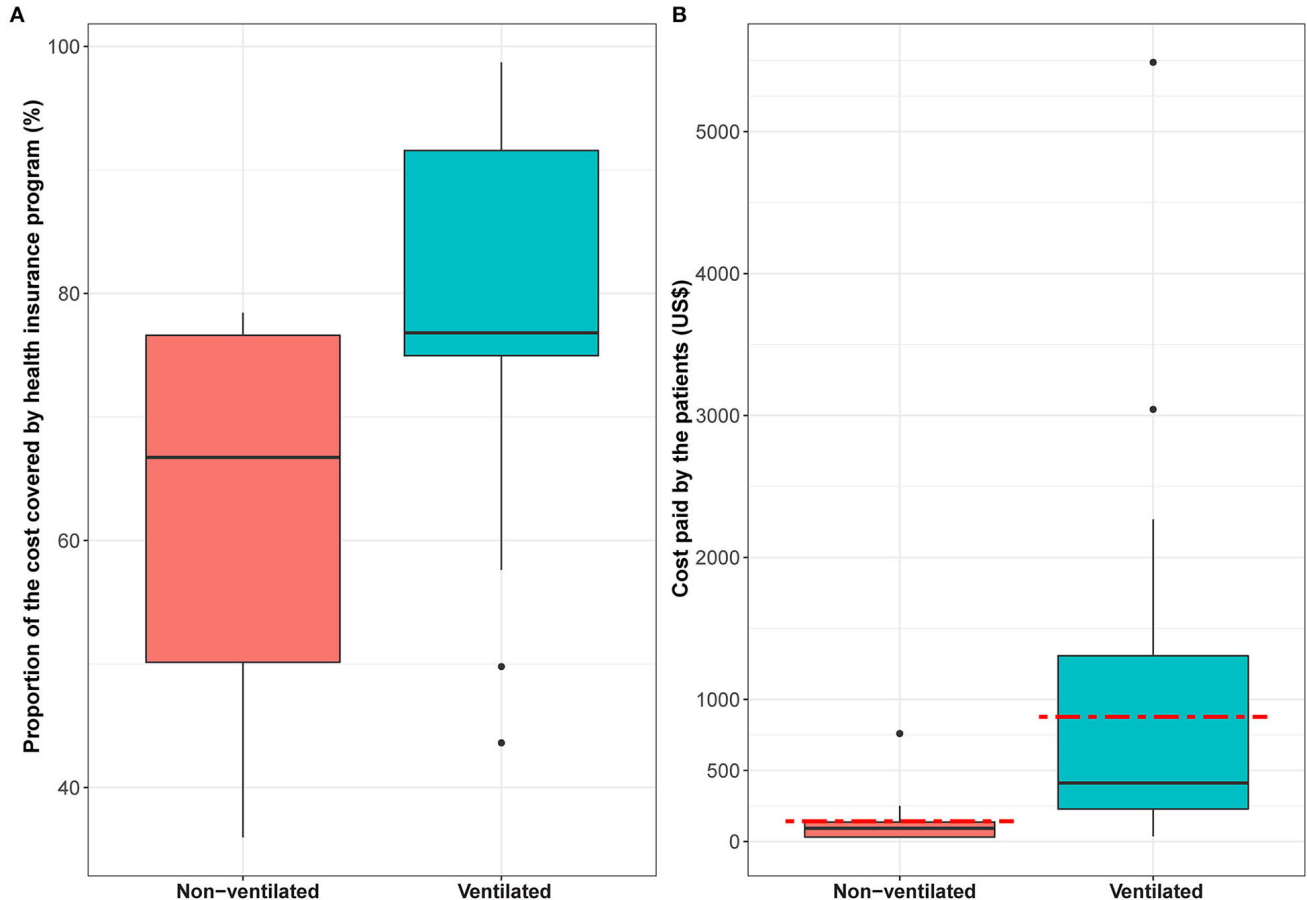
The ICU costs covered by the national health insurance program. Panel **(A)** shows for the patients enrolled in the national health insurance program, the proportion of their total ICU costs that were covered. Panel **(B)** shows for the patients enrolled in the national health insurance program the ICU costs (US$) paid by the patients. The box plot illustrates the interquartile range, the black line represents the median and the red dashed line represents the mean. Costs are in US$ 2019 prices.

Among our sample, 70% of the patients with dengue and sepsis were covered by the national health insurance program (no health insurance data were available for the patients with tetanus as this data was not collected within the primary study where patients costs were covered by the clinical trial) (Table 2) (35). For these insured patients, the median proportion of their total cost that was covered by the health insurance program was 76.4% (IQR 68.8, 78.5%). Interestingly, while the median proportion of ICU costs covered by the insurance program for those who did not require mechanical ventilation was only 66.7%, it was nearly 77% for the patients who required mechanical ventilation - despite their status being more severe, and them requiring more medication and other services (Figure 2A). Despite the national health insurance covering a large proportion of the ICU costs, the mean remaining portion that would be covered by the patients was US$142 (min-max: US$27.9–759.7) and US$877 (min-max: US$35.2–5,487.6) for patients without and with mechanical ventilation, respectively (Figure 2B).

## Discussion

This study focused on the direct medical costs incurred by infectious disease patients staying in the ICU of a tertiary hospital in HCMC. We found that ICU care is associated with significant direct medical costs, particularly for patients requiring mechanical ventilation and that the disease and associated severity are the main cost drivers of ICU care. The total cost was also highly influenced by the duration of ICU stay, with a longer stay associated with more severe disease and the possibility of complications and hospital-acquired infections.

This study demonstrates the notable cost of ICU care in Vietnam and similar LMIC settings and highlights the importance of evaluating novel critical care interventions that could reduce the costs of ICU care in such settings (even if they only have little benefit regarding improving mortality outcomes). The specific costs we estimated will be useful for the evaluation of interventions to control the specific diseases investigated, including not only critical care interventions but also population level interventions. For example, in the case of dengue, it could be used within the economic evaluation of vaccines or Wolbachia deployments. In tetanus, this data could be used in the justification of strengthening vaccination programs, particularly of at at-risk groups. Similarly, this data could be used in the evaluation of novel vaccines for causes of sepsis. Furthermore, as the main cost drivers of ICU care were found to be disease and associated severity, our results imply that general critical care cost estimates can be misleading/inaccurate when evaluating a particular disease. This variation in cost drivers needs to be considered when comparing the results of different costing studies. Finally, this data highlights the vital need for future studies to further evaluate the costs and economic burden incurred by ICU patients in LMIC settings. This should include further examination of the implications of these critical care costs on different aspects of society, such as healthcare affordability, patients' attitudes and equity.

The median ICU length of stay in this study (13 days) was longer than reported in other studies in Southeast Asia (typically a median of 4–6.4 days) (14, 16, 36, 37). This may be due to differences in the case mix, the type of ICU investigated (i.e., a general ICU vs. an infectious disease specific ICU) and the eligibility criteria in our primary studies (Supplementary Figure 1).

In general, medication costs were the main cost driver for patients irrespective of mechanical ventilation requirement. Not surprisingly, with the exception of sepsis cases, for patients not requiring ventilation, the costs related to antibiotics were trivial in the case of dengue (a viral disease not requiring antibiotics) and tetanus (where inexpensive antibiotics are first-line treatment). However, the costs for antibiotics were significant for all sampled patients requiring mechanical ventilation. This is likely due to ventilator-associated respiratory infections, other hospital-acquired infections and possible diagnostic uncertainty in severe patients necessitating empiric antibiotic therapy (32, 36, 38). These patients also received expensive ‘last-line' antibiotics (such as carbapenems) due to high levels of antibiotic resistance (32, 36, 38). Thus, preventing hospital-acquired infection, identifying the need for, and safely reducing the duration of antibiotic therapy should be priorities for novel critical care interventions.

There are limited costing studies in Vietnam to compare these estimates to. Our total hospital admission cost estimate for non-ventilated dengue patients that needed the ICU (US$150) was similar to previous estimates regarding the cost of hospitalized dengue patients in Vietnam (which ranged from US$46-153 – 2019 prices) (39). Note that our estimates would be expected to be higher as they only relate to patients needing the ICU, whereas the other estimates would include patients only needing care in the general ward (39). Our calculated cost for patients with dengue requiring mechanical ventilation (US$4,250) was significantly higher than this range and this highlights that some hospitalized dengue patients do experience much higher costs than the averages typically considered because clinically dengue varies from a relatively mild disease to one causing severe multi-organ failure (25).

This study used the available data from previous trials conducted at the Hospital for Tropical Diseases in HCMC. Although it did not follow any established protocol the bottom-up approach employed is commonly used and generally believed to be the preferred methodology for such a costing because it values each cost component for the individual patients (13).

The direct medical cost estimates of ICU care in our study were significantly lower than those for Thailand, Malaysia and China, with corresponding medians of US$2,716, US$5,207 and US$18,889 per ICU stay, respectively (14–16). These costs are not directly comparable due to differences in the diseases investigated, methods employed (top-down vs bottom-up approaches and types of costs included) and cost year of the studies. However, there were still some similarities to these other studies. For instance, the costs related to medications were also reported to be a dominant cost category in the study by Ye et al. (15). Moreover, we found that the ICU costs of those who used mechanical ventilation were significantly higher than those that did not, similar to results by Khwannimit et al. (16). Many studies in high-income countries have suggested that mechanical ventilation increases the cost of critical care (12, 13, 40). This may be for several reasons: either it is an indicator of more severely ill patients likely to require more treatment for longer periods of time but also as it often requires a higher level of staff monitoring and facility costs such as oxygen (12, 13, 40). In addition, a study estimating costs across seven ICU departments in Germany, Italy, the Netherlands and United Kingdom found that the average direct cost per ICU day ranged from €1,168 to €2,025 (13). This is notably higher than the daily ICU costs we estimated (with the average ranging between US$33-406). They also highlight the importance of the patient case-mix when comparing cost differences between ICU departments.

Importantly, the total direct medical cost associated with ICU care for non-ventilated and ventilated patients in our study was 1.9 and 19.1 times higher than Vietnam's annual average per capita health care spending in 2018 (US$134.3, 2018 prices), respectively (41). Hence, these costs are significant for this setting, even though they are smaller than the costs found in other countries (14–16). It should also be recognized that tetanus is a vaccine-preventable disease, where an inexpensive effective vaccine is readily available.

The national health insurance program in Vietnam aims to support financing and access of health care services, and to reduce patients' out-of-pocket healthcare costs, particularly for the poor and vulnerable populations. For those within our sample covered by the national health insurance program, the median proportion of their total ICU costs covered by the program was 76.4% (IQR 68.8, 78.5%). Despite this high level of coverage, the remaining portion of the costs that patients paid was equivalent to a mean of US$142.4 and US$877.4 for non-ventilated patients and ventilated patients, respectively (Figure 2). These mean costs were 0.8 times and 4.9 times higher than the 2019 monthly minimum wage (US$180) (42) for urban Hanoi and HCMC (which is higher than the minimum wage for other areas in Vietnam). It should be noted that the majority of patients admitted to the Hospital for Tropical Diseases came from provinces outside of HCMC. This meant that the patients' status was commonly more severe, and their income was lower than the average in HCMC. Although the health insurance coverage of the poor and in urban areas is high, the coverage rate of the near-poor and those in the informal sectors (such as farmers) is low (22). Overall, ICU care still causes a high economic burden on patients, even for those that have health insurance and although the national health insurance program is designed for financial protection, patients still incur notable out-of-pocket payments (25).

Further investigation of strategies to lower ICU costs is needed, particularly in low-resource settings such as Vietnam. This will require the consideration of multiple aspects of healthcare, including medical research/innovations, collaboration with other actors/stakeholders (such as equipment manufacturers and training programs), as well as monitoring and assessment. The use of management frameworks/theory [such as Vuong et al. (43)] could have a role in accounting for these different aspects. Technological innovations could also play a role in reducing these ICU costs. It will be important to consider the perceptions of science in the government, institutions and the public when implementing new technological innovations (44).

### Limitations

This study used a sample of ICU patients' medical bills that were retrieved from three different studies at the Hospital for Tropical Diseases in HCMC (the eligibility criteria for these studies is outlined in Supplementary Figure 1). Although it is difficult to assess exactly how representative the overall sample is to general ICU admissions for these diseases, the primary studies had very clearly defined inclusion criteria and screening logs compared to observational cohorts. In addition, the inclusion criteria of the primary studies reflected the need to be generalizable to other settings.

Our aim in this study was to investigate the direct medical costs for locally important infectious diseases in Vietnam. However, in using data from a tertiary referral hospital in HCMC it is possible that patients were generally more severe compared to other ICUs. To overcome this, we stratified for mechanical ventilation status and aimed to provide an approximate indicator of disease severity. Consequently, our sample sizes for certain subgroups for dengue and sepsis cases were small, introducing uncertainty in the exact cost estimates. In addition, due to the variation in ICU costs, it is also likely that our results provide only an approximate estimate of ICU costs for other diseases, settings and patient populations in Vietnam. This also highlights the need for further studies investigating ICU costs, with larger samples and including more diseases and study settings. That said, although there might be some variation in the unit costs for treating these diseases in other hospitals within Vietnam outside of HCMC, the overall conclusions that ICU care results in significant direct medical costs (particularly for patients requiring mechanical ventilation) and that the main cost drivers of ICU care depend on the disease and associated severity, would be robust to this.

The cost data estimates calculated within this paper were based directly on the charges from the patients' hospital bills and the costs related to the staff time were assumed to be captured by the charges for the different services. However, these charges do not necessarily reflect the economic value of the resources utilized for their care (45–47). In order to try and capture economic costs within this context, a cost-to-charge ratio is commonly applied to the charges (which is based on the ratio of hospital's (or department's) expenses and what they charge) (48, 49) but the data were not available to do this adjustment within this study.

## Conclusion

To conclude, ICU care in the Hospital for Tropical Diseases in HCMC results in substantial direct medical costs, particularly for patients requiring mechanical ventilation. The main cost drivers of ICU care depend on the disease and associated severity. Understanding the burden of ICU cost is crucial for informing and supporting decision-making, particularly in LMIC, such as Vietnam. Further studies are needed to fully evaluate the costs and economic burden incurred by ICU patients. Understanding the main drivers cost in ICU is important for policymakers and hospital managers and will be vital for the evaluation of novel interventions/technology to improve critical care in LMICs.

## Data Availability Statement

The data analyzed in this study are available on reasonable request. Requests to access the datasets should be directed to TH, hungtm@oucru.org.

## Ethics Statement

The primary studies involving human participants were reviewed and approved by the Hospital for Tropical Disease (Ho Chi Minh City) Ethics Committee and Oxford Tropical Research Ethics Committee. The patients/participants provided their written informed consent to participate in this study.

## Members of Vietnam ICU Translational Applications Laboratory (VITAL) investigators

OUCRU inclusive authorship list in Vietnam (alphabetic order by surname): Dang Phuong Thao, Dang Trung Kien, Doan Bui Xuan Thy, Dong Huu Khanh Trinh, Du Hong Duc, Ronald Geskus, Ho Bich Hai, Ho Quang Chanh, Ho Van Hien, Huynh Trung Trieu, Evelyne Kestelyn, Le Dinh Van Khoa, Le Thanh Phuong, Luu Hoai Bao Tran, Luu Phuoc An, Angela Mcbride, Nguyen Lam Vuong, Nguyen Quang Huy, Nguyen Than Ha Quyen, Nguyen Thanh Ngoc, Nguyen Thi Giang, Nguyen Thi Le Thanh, Nguyen Thi Phuong Dung, Nguyen Thi Phuong Thao, Ninh Thi Thanh Van, Phan Nguyen Quoc Khanh, Phung Khanh Lam, Phung Tran Huy Nhat, Guy Thwaites, Tran Minh Duc, Jennifer Ilo Van Nuil, Vu Ngo Thanh Huyen, Hospital for Tropical Diseases, Ho Chi Minh City (alphabetic order by surname): Cao Thi Tam, Duong Bich Thuy, Ha Thi Hai Duong, Ho Dang Trung Nghia, Le Buu Chau, Le Mau Toan, Le Ngoc Minh Thu, Le Thi Mai Thao, Luong Thi Hue Tai, Nguyen Hoan Phu, Nguyen Quoc Viet, Nguyen Thanh Nguyen, Nguyen Thi Kim Anh, Nguyen Van Thanh Duoc, Pham Kieu Nguyet Oanh, Phan Thi Hong Van, Phan Tu Qui, Phan Vinh Tho, Truong Thi Phuong Thao. University of Oxford (alphabetic order by surname): Natasha Ali, David Clifton, Mike English, Shadi Ghiasi, Heloise Greeff, Jannis Hagenah, Ping Lu, Jacob McKnight, Chris Paton, Tingting Zhu Imperial College London (alphabetic order by surname): Pantelis Georgiou, Bernard Hernandez Perez, Kerri Hill-Cawthorne, Alison Holmes, Stefan Karolcik, Damien Ming, Nicolas Moser, Jesus Rodriguez Manzano King's College London (alphabetic order by surname): Liane Canas, Alberto Gomez, Hamideh Kerdegari, Marc Modat, Reza Razavi, Miguel Xochicale University of Ulm (alphabetic order by surname): Walter Karlen The University of Melbourne (alphabetic order by surname): Linda Denehy, Thomas Rollinson Mahidol Oxford Tropical Medicine Research Unit (MORU) (alphabetic order by surname): Luigi Pisani, Marcus Schultz.

## Author Contributions

TH and HT conceived the study. TH entered, cleaned the data, and wrote the first draft of the paper. NV, LY, AM, VD, HL, NP, CT, HD, BN, and NVVC collected and contributed the data for the analysis. All authors contributed to the content and writing of the paper, read and approve the final of manuscript.

## Funding

This study was supported by the Wellcome Trust VITAL project grant 217650/Z/19/Z. The primary studies from which the sample was taken from were supported by a Wellcome Trust fellowship (107367/Z/15/Z) to CT, a Wellcome Trust Ph.D. Fellowship award 203905/Z/16/Z to AM and funding from the OUCRU Wellcome Trust core grant 106680/B/14/Z. HCT acknowledges funding from the MRC Centre for Global Infectious Disease Analysis (reference MR/R015600/1), jointly funded by the UK Medical Research Council (MRC) and the UK Foreign, Commonwealth & Development Office (FCDO), under the MRC/FCDO Concordat agreement and is also part of the EDCTP2 programme supported by the European Union. Funders have no roles in study design, collection, analysis, and decision to publish or preparation of the manuscript. For the purpose of open access, the author has applied a ‘Creative Commons Attribution (CC BY) licence to any Author Accepted Manuscript version arising from this submission.

## Conflict of Interest

The authors declare that the research was conducted in the absence of any commercial or financial relationships that could be construed as a potential conflict of interest.

## Publisher's Note

All claims expressed in this article are solely those of the authors and do not necessarily represent those of their affiliated organizations, or those of the publisher, the editors and the reviewers. Any product that may be evaluated in this article, or claim that may be made by its manufacturer, is not guaranteed or endorsed by the publisher.

## References

[1] Haniffa R De Silva AP de Azevedo L Baranage D Rashan A Baelani I. Improving ICU services in resource-limited settings: perceptions of ICU workers from low-middle-, and high-income countries. J Crit Care. (2018) 44:352–6. 10.1016/j.jcrc.2017.12.007 29275269

[2] Baelani I Jochberger S Laimer T Otieno D Kabutu J Wilson I. Availability of critical care resources to treat patients with severe sepsis or septic shock in Africa: a self-reported, continent-wide survey of anaesthesia providers. Crit Care. (2011) 15:R10. 10.1186/cc9410 21219619 PMC3222039

[3] Murthy S Leligdowicz A Adhikari NK. Intensive care unit capacity in low-income countries: a systematic review. PLoS ONE. (2015) 10:e0116949. 10.1371/journal.pone.0116949 25617837 PMC4305307

[4] Kwizera A DA Dr M Nakibuuka J. National intensive care unit bed capacity and ICU patient characteristics in a low income country. BMC Res Notes. (2012) 5:475. 10.1186/1756-0500-5-475 22937769 PMC3470976

[5] Phua J Faruq MO Kulkarni AP Redjeki IS Detleuxay K Mendsaikhan N. Critical care bed capacity in Asian countries and regions. Crit Care Med. (2020) 48:654–62. 10.1097/CCM.0000000000004222 31923030

[6] Rhodes A Ferdinande P Flaatten H Guidet B Metnitz PG Moreno RP. The variability of critical care bed numbers in Europe. Intensive Care Med. (2012) 38:1647–53. 10.1007/s00134-012-2627-8 22777516

[7] Gooch RA Kahn JM. ICU bed supply, utilization, and health care spending: an example of demand elasticity. JAMA. (2014) 311:567–8. 10.1001/jama.2013.283800 24408679

[8] Turner HC Hao NV Yacoub S Hoang VMT Clifton DA Thwaites GE. Achieving affordable critical care in low-income and middle-income countries. BMJ Global Health. (2019) 4:e001675. 10.1136/bmjgh-2019-001675 31297248 PMC6590958

[9] Halpern NA Pastores SM. Critical care medicine beds, use, occupancy, and costs in the United States: a methodological review. Crit Care Med. (2015) 43:2452–9. 10.1097/CCM.0000000000001227 26308432 PMC5520980

[10] Coopersmith CM Wunsch H Fink MP Linde-Zwirble WT Olsen KM Sommers MS. A comparison of critical care research funding and the financial burden of critical illness in the United States. Crit Care Med. (2012) 40:1072–9. 10.1097/CCM.0b013e31823c8d03 22202712

[11] Halpern NA Pastores SM. Critical care medicine in the United States 2000-2005: an analysis of bed numbers, occupancy rates, payer mix, and costs. Crit Care Med. (2010) 38:65–71. 10.1097/CCM.0b013e3181b090d0 19730257

[12] Kaier K Heister T Wolff J Wolkewitz M. Mechanical ventilation and the daily cost of ICU care. BMC Health Serv Res. (2020) 20:1–5. 10.1186/s12913-020-05133-5 32234048 PMC7106643

[13] Tan SS Bakker J Hoogendoorn ME Kapila A Martin J Pezzi A. Direct cost analysis of intensive care unit stay in four European countries: applying a standardized costing methodology. Value Health. (2012) 15:81–6. 10.1016/j.jval.2011.09.007 22264975

[14] Aung YN Nur AM Ismail A Aljunid SM. Determining the cost and length of stay at intensive care units and the factors influencing them in a teaching hospital in Malaysia. Value Health Reg Issues. (2020) 21:149–56. 10.1016/j.vhri.2019.09.006 31958748

[15] Ye Y Zhu B Jiang L Jiang Q Wang M Hua L. A contemporary assessment of acute mechanical ventilation in Beijing: description, costs, and outcomes. Crit Care Med. (2017) 45:1160–7. 10.1097/CCM.0000000000002360 28422775 PMC5470868

[16] Khwannimit B Bhurayanontachai R. The direct costs of intensive care management and risk factors for financial burden of patients with severe sepsis and septic shock. J Crit Care. (2015) 30:929–34. 10.1016/j.jcrc.2015.05.011 26051981

[17] Peter JV Thomas K Jeyaseelan L Yadav B Sudarsan TI Christina J. Cost of Intensive care in India. Int J Technol Assess Health Care. (2016) 32:241–5. 10.1017/S0266462316000398 27608529

[18] The World Bank. Current health expenditure per capita (current US$) - China, Malaysia, Thailand, India, Vietnam 2021 [accessed December 6, 2021]. Available online at: https://data.worldbank.org/indicator/SH.XPD.CHEX.PC.CD?end=2018&locations=CN-MY-TH-IN-VN&start=2017

[19] Thuy DB Campbell J Nhat LTH Hoang NVM Hao NV Baker S. Hospital-acquired colonization and infections in a Vietnamese intensive care unit. PLoS ONE. (2018) 13:e0203600. 10.1371/journal.pone.0203600 30192894 PMC6128614

[20] Vu DP. Burden, Etiology and Control of Hospital Acquired Infections in Intensive Care Units in Vietnam: The Open University (2017). 10.21954/ou.ro.0000c5f1

[21] Duc Thanh N Anh BTM Thanh Hung P Quynh Anh P Huyen Xiem C. Impact of public health insurance on out-of-pocket health expenditures of the near-poor in Vietnam. Health Serv Insights. (2021) 14:11786329211017411. 10.1177/11786329211017411 34093020 PMC8142235

[22] Thuong NTT. Impact of health insurance on healthcare utilization patterns in Vietnam: a survey-based analysis with propensity score matching method. BMJ Open. (2020) 10:e040062. 10.1136/bmjopen-2020-040062 33046477 PMC7552874

[23] Thuong NTT Huy TQ Tai DA Kien TN. Impact of health insurance on health care utilisation and out-of-pocket health expenditure in Vietnam. BioMed Res Int. (2020) 2020:9065287. 10.1155/2020/9065287 32908923 PMC7471796

[24] Nguyen KT Khuat OTH Ma S Pham DC Khuat GTH Ruger JP. Impact of health insurance on health care treatment and cost in Vietnam: a health capability approach to financial protection. Am J Public Health. (2012) 102:1450–61. 10.2105/AJPH.2011.300618 22698046 PMC3464830

[25] McBride A Thuy Duong B Chau Nguyen VV Thwaites CL Turner HC Hao Nguyen V. Catastrophic health care expenditure due to septic shock and dengue shock in Vietnam. Trans R Soc Trop Med Hyg. (2019) 113:649–51. 10.1093/trstmh/trz064 31340045 PMC6792161

[26] Vuong QH. Be rich or don't be sick: estimating Vietnamese patients' risk of falling into destitution. Springerplus. (2015) 4:529. 10.1186/s40064-015-1279-x 26413435 PMC4577521

[27] Loan HT Yen LM Kestelyn E Hao NV Thanh TT Dung NTP. Intrathecal Immunoglobulin for treatment of adult patients with tetanus: a randomized controlled 2x2 factorial trial. Wellcome Open Res. (2018) 3:58. 10.12688/wellcomeopenres.14587.2 30809591 PMC6372971

[28] Dat VQ Geskus RB Wolbers M Loan HT Yen LM Binh NT. Continuous versus intermittent endotracheal cuff pressure control for the prevention of ventilator-associated respiratory infections in Vietnam: study protocol for a randomised controlled trial. Trials. (2018) 19:217. 10.1186/s13063-018-2587-6 29615093 PMC5883270

[29] Dat VQ Yen LM Loan HT Phu VD Binh NT Geskus RB. Effectiveness of continuous endotracheal cuff pressure control for the prevention of ventilator associated respiratory infections: an open-label randomized, controlled trial. Clin Infect Dis. (2021) ciab724. 10.1093/cid/ciab724 34420048 PMC9155610

[30] Ministry of Health. Decision 3705/QD-BYT of Ministry of Healh Issuing Guidelines on Clinical Diagnosis and Treatment of Dengue. Hanoi: Ministry of Health (2019).

[31] Ministry of Health. Decision 5642/QD-BYT of Ministry of Health Issuing Guidelines on Clinical Diagnosis and Treatment of Infectious Diseases. Hanoi: Ministry of Health (2015).

[32] Phu VD Nadjm B Duy NHA Co DX Mai NTH Trinh DT. Ventilator-associated respiratory infection in a resource-restricted setting: impact and etiology. J Intensive Care. (2017) 5:69. 10.1186/s40560-017-0266-4 29276607 PMC5738227

[33] International Monetary Fund. Vietnam: Gross domestic product, deflator (Index) 2020 Available online at: https://www.imf.org/en/Publications/WEO/weo-database/2018/October/weo-report?c=582,&s=NGDP_D,&sy=2010&ey=2021&ssm=0&scsm=1&scc=0&ssd=1&ssc=0&sic=0&sort=country&ds=.&br=1

[34] World Bank. Official exchange rate (LCU per US$, period average) - Vietnam 2020 (accessed July 23, 2020). Available online at: https://data.worldbank.org/indicator/PA.NUS.FCRF?locations=VN

[35] Van Hao N Loan HT Yen LM Kestelyn E Hong DD Thuy DB. Human vs. equine intramuscular antitoxin, with or without human intrathecal antitoxin, for the treatment of adults with tetanus: a 2x2 factorial randomised controlled trial. Lancet Glob Health. (2022) 10:e8621e72. 10.1016/S2214-109X(22)00117-6 35561721 PMC9115864

[36] Phu VD Wertheim HFL Larsson M Nadjm B Dinh Q-D Nilsson LE. Burden of hospital acquired infections and antimicrobial use in Vietnamese adult intensive care units. PLoS ONE. (2016) 11:e0147544. 10.1371/journal.pone.0147544 26824228 PMC4732823

[37] Vincent J-L Marshall JC Ñincent -Silva SA Franrshall JC red Loeches I Lipman J. Assessment of the worldwide burden of critical illness: the intensive care over nations (ICON) audit. Lancet Respir Med. (2014) 2:380–6. 10.1016/S2213-2600(14)70061-X 24740011

[38] Dat VQ Huong VTL Turner HC Thwaites L van Doorn HR Nadjm B. Excess direct hospital cost of treating adult patients with ventilator associated respiratory infection (VARI) in Vietnam. PLoS ONE. (2018) 13:e0206760. 10.1371/journal.pone.0206760 30379956 PMC6209379

[39] Hung TM Clapham HE Bettis AA Cuong HQ Thwaites GE Wills BA. The estimates of the health and economic burden of dengue in Vietnam. Trends Parasitol. (2018) 34:1266–71. 10.1016/j.pt.2018.07.007 30100203 PMC6192036

[40] Dasta JF McLaughlin TP Mody SH Piech CT. Daily cost of an intensive care unit day: the contribution of mechanical ventilation. Crit Care Med. (2005) 33:1266–71. 10.1097/01.CCM.0000164543.14619.00 15942342

[41] Institute for Health and Metrics and Evaluation (IHME). Financing Global Health 2018: Countries and Program in Transitions. Seattle, WA: IHME (2019).

[42] Vietnam government. Decree No. 90/2019/Nd-CP Region-Based Minimum Wage Levels Applicable to Employees Working Under Labor Contracts. Hanoi: Vietnam Gorvernment (2019).

[43] Vuong QH Le TT La VP Nguyen HTT Ho MT Van Khuc Q. Covid-19 vaccines production and societal immunization under the serendipity-mindsponge-3D knowledge management theory and conceptual framework. Humanit Soc Sci Commun. (2022) 9:22. 10.1057/s41599-022-01034-6

[44] Vuong QH. The (ir)rational consideration of the cost of science in transition economies. Nat Hum Behav. (2018) 2:5. 10.1038/s41562-017-0281-4 30980055

[45] Sandmann FG Robotham JV Deeny SR Edmunds WJ Jit M. Estimating the opportunity costs of bedbdays. Health Econ. (2018) 27:592–605. 10.1002/hec.3613 29105894 PMC5900745

[46] Shwartz M Young DW Siegrist R. The ratio of costs to charges: how good a basis for estimating costs? Inquiry. (1995) 32:476–81. 8567084

[47] Finkler SA. The distinction between cost and charges. Ann Intern Med. (1982) 96:102rn M 10.7326/0003-4819-96-1-102 7053682

[48] Nguyen T-P-L Nguyen TBY Nguyen TT Vinh Hac V Le HH Schuiling-Veninga CCM. Direct costs of hypertensive patients admitted to hospital in Vietnam' a bottom-up micro-costing analysis. BMC Health Serv Res. (2014) 14:514. 10.1186/s12913-014-0514-4 25348043 PMC4221683

[49] Anh DD Riewpaiboon A Tho LH Kim SA Nyambat B Kilgore P. Treatment costs of pneumonia, meningitis, sepsis, and other diseases among hospitalized children in Viet nam. J Health Pop Nutr. (2010) 28:436–42. 10.3329/jhpn.v28i5.6151 20941894 PMC2963765

