# Supplementary material for: Direct Medical Costs of Tetanus, Dengue, and Sepsis Patients in an Intensive Care Unit in Vietnam
Source: Front Public Health. 2022 Jun 20;10:893200. doi: 10.3389/fpubh.2022.893200 (PMC9263973; doi:10.3389/fpubh.2022.893200)
Supplement: Supplementary file 1 [file Data_Sheet_1.PDF]

Supplementary information

Supplementary Table S1: General background information

	Dengue (N=28)	Sepsis (N=42)	Tetanus (N=272)	Overall (N=342)
**Gender** n(%)				
Female	17 (60.7%)	12 (28.6%)	43 (15.8%)	72 (21.1%)
Male	11 (39.3%)	30 (71.4%)	229 (84.2%)	270 (78.9%)
**Address** n(%)				
Ho Chi Minh	12 (42.9%)	15 (35.7%)	53 (19.5%)	80 (23.4%)
Other provinces	16 (57.1%)	27 (64.3%)	219 (80.5%)	262 (76.6%)
**Age**				
Mean (SD)	32.2 (14.0)	52.7 (16.3)	49.1 (14.4)	48.1 (15.4)
**Health insurance** n(%)				
No	9 (32.1%)	12 (28.6%)	0 (0)	21 (6.1%)
Yes	19 (67.9%)	30 (71.4%)	0 (0%)	49 (14.3%)
Not available	0 (0%)	0 (0%)	272 (100%)	272 (79.5%)
**Number of hospital days**				
Mean (SD)	8.32 (6.57)	17.9 (14.0)	26.3 (16.5)	23.8 (16.5)
Median (Q1, Q3)	6.0 (4.0, 10.3)	15.5 (6.75, 23.8)	23.5 (17.0, 30.0)	22.0 (15.0, 29.0)
**Number of ICU days**				
Mean (SD)	6.0 (5.87)	13.2 (11.2)	17.7 (16.2)	16.2 (15.4)
Median (Q1, Q3)	3.5 (2.0, 8.5)	10.0 (5.3, 15.8)	15.0 (8.0, 23.0)	13.0 (7.0, 22.0)

SD: Standard deviation, Q1: 25% quartile and Q3: 75% quartile

Supplementary Table S2: The antibiotic costs relating to the ICU patients

Median cost (US$)	Dengue		Sepsis		Tetanus	
	No mechanical ventilation [N=15]	Received mechanical ventilation [N=13]	No mechanical ventilation [N=6]	Received mechanical ventilation [N=36]	No mechanical ventilation [N=141]	Received mechanical ventilation [N=131]
**Median total antibiotic cost (Q1, Q3)**	0 (0;0)	267 (146; 616)	172 (155; 233)	415 (160; 783)	0.2(0.2; 0.3)	75 (0.3; 329)
**Median daily ICU antibiotic cost (Q1, Q3)**	0 (0, 0)	41.1 (18.4, 52.8)	36.1 (21.4, 47.0)	37.9 (13.1, 62.6)	0 (0, 0)	2.7 (0, 12.3)

Costs are in US$ 2019 prices. Q1: 25% quartile and Q3: 75% quartile

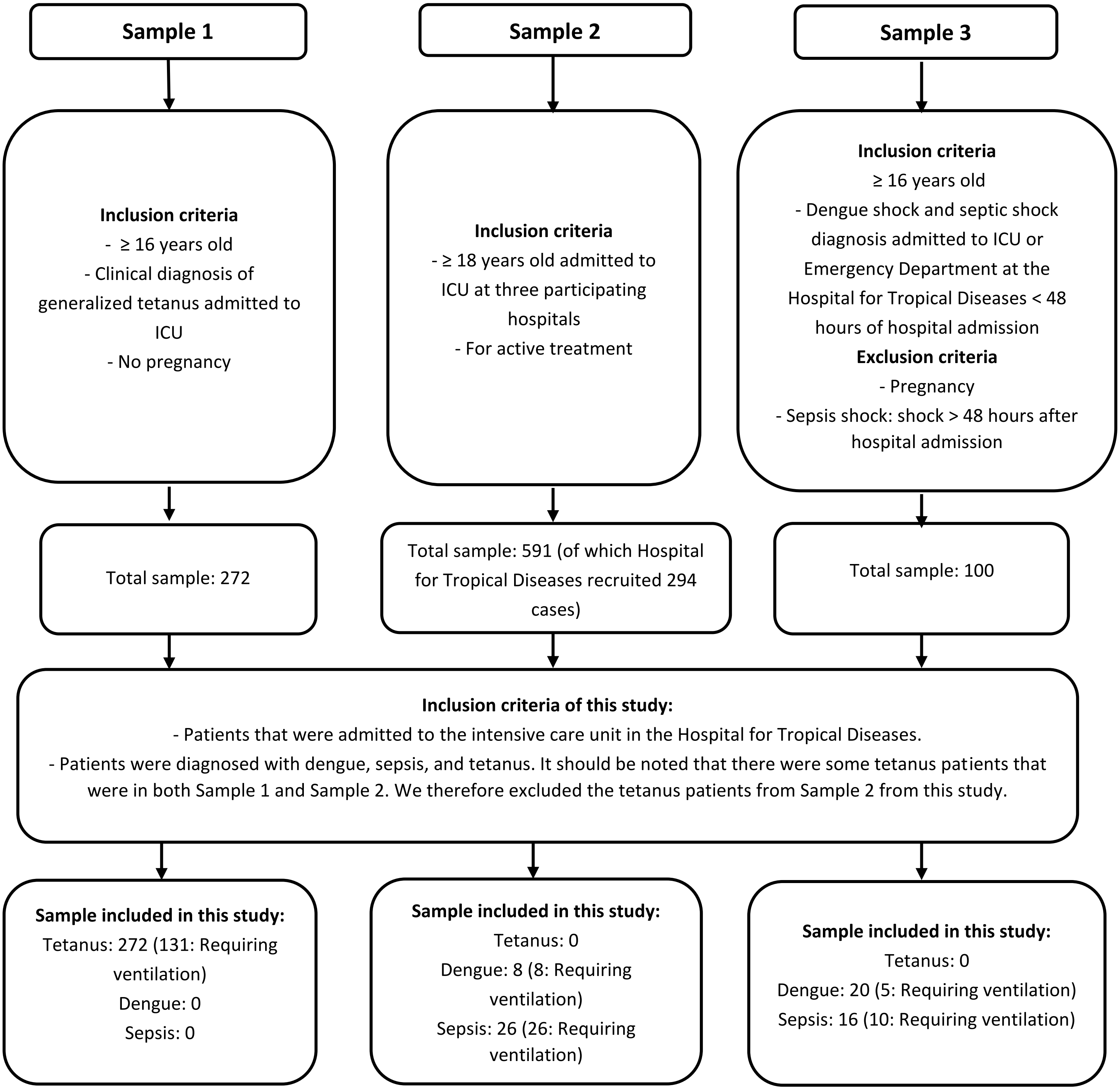

Supplementary Figure S1: Flow-chart outlining the sample selection. *Further detail regarding the primary studies from which Sample 1 and Sample 2 were taken are reported in Loan et al. (1), Dat et al. (2) respectively. Details regarding the study related to Sample 3 are not currently published.*

Supplementary Box 1: Background information on the diseases investigated

Dengue is a seasonal viral disease that causes substantial health and economic burden in Vietnam (3). The Global Burden of Disease (GBD) study estimated that there were 1,038,967 dengue symptomatic cases in Vietnam in 2019 (with a 95% uncertainty interval (UI) of 812,230-1,333,874) (4). Generally, 95% of dengue cases are mild and moderate, and around 5% of hospitalized cases progress to severe disease (5). While most of the cases can be treated at any district level hospital, severe cases (such as those with dengue shock syndrome), can require intensive care and often need to be transferred to higher level hospitals. It should be noted that currently there is no specific treatment for dengue other than supportive measures (6), thus early identification of the severe manifestations of the disease and the experience of physicians play an important role in treating the patients. The following references provide the latest treatment guidelines for dengue (7, 8).

Tetanus is a vaccine-preventable bacterial disease that still commonly occurs in many low-income and middle-income countries. In Vietnam, there are currently two programs that help to prevent tetanus, the expanded Program on Immunization (EPI) and the Maternal and Neonatal Tetanus (MNT) initiative. Whilst the MNT has achieved notable success, the tetanus prevention program's impact in terms of preventing non-neonatal cases is unknown (9). There is currently no exact estimate of the incidence of tetanus in Vietnam. The number of tetanus patients admitted to Hospital for Tropical Diseases in Ho Chi Minh City varied from 250-350 patients treated annually (10). Treatment for tetanus aims to control muscle spasms, prevent toxin uptake and provide supportive care (11). Without mechanical ventilation support, the risk of mortality is high and even with such care, patients require several weeks of hospitalization (11). The following reference provides the latest treatment guidelines for tetanus in Vietnam (12).

In contrast to dengue and tetanus, sepsis is the presence of systemic host responses to infection. Severe sepsis and septic shock are considered major healthcare problems and kill millions of people annually worldwide (13). In 2017, it was estimated that there were 312,868 sepsis cases in Vietnam (95%UI: 251,070–398,503). Among those, 187,134, 105,880, and 19,854 cases were related to infections, non-communicable diseases and injury, respectively (14). The most common pathogens found in Vietnam causing sepsis are gram-negative bacteria, such as Klebsiella Pneumonia, Escherichia Coli and Acinetobacter Baumannii (15-17). Monitoring sepsis and sepsis shock are commonly based on clinical symptoms and laboratory results. It is therefore challenging to diagnose and manage even in ICUs in high-income countries (18). The following reference provides the latest treatment guidelines for sepsis in Vietnam (12).

References

1. Loan HT, Yen LM, Kestelyn E, Hao NV, Thanh TT, Dung NTP, et al. Intrathecal Immunoglobulin for treatment of adult patients with tetanus: A randomized controlled 2x2 factorial trial. Wellcome Open Res. 2018;3:58.
2. Dat VQ, Geskus RB, Wolbers M, Loan HT, Yen LM, Binh NT, et al. Continuous versus intermittent endotracheal cuff pressure control for the prevention of ventilator-associated respiratory infections in Vietnam: study protocol for a randomised controlled trial. Trials. 2018;19(1):217-.
3. Hung TM, Clapham HE, Bettis AA, Cuong HQ, Thwaites GE, Wills BA, et al. The estimates of the health and economic burden of dengue in Vietnam. Trends Parasitol. 2018;34(10):904-18.
4. IHME. GBD Results Tools 2019 [Available from: http://ghdx.healthdata.org/gbd-results-tool.
5. Salomon JA, Haagsma JA, Davis A, de Noordhout CM, Polinder S, Havelaar AH, et al. Disability weights for the Global Burden of Disease 2013 study. Lancet Glob Health. 2015;3:e712-e23.
6. World Health Organization. Dengue and Severe Dengue 2019 [cited 2019 25 Nov]. Available from: https://www.who.int/news-room/fact-sheets/detail/dengue-and-severe-dengue.
7. Yacoub S, Wills B. Dengue: an update for clinicians working in non-endemic areas. Clin Med (Lond). 2015;15(1):82-5.
8. Ministry of Health. Quyết định 3705/QĐ-BYT của Bộ Y Tế ban hành hướng dẫn chẩn đoán, điều trị sốt xuất huyết. 2019.
9. Jit M, Huyen DTT, Friberg I, Van Minh H, Kiet PHT, Walker N, et al. Thirty years of vaccination in Vietnam: impact and cost-effectiveness of the national expanded programme on immunization. Vaccine. 2015;33:A233-A9.
10. Hao NV, Yen LM, Davies-Foote R, Trung TN, Duoc NVT, Trang VTN, et al. The management of tetanus in adults in an intensive care unit in Southern Vietnam. Wellcome Open Res. 2021;6:107.
11. Yen L, Thwaites C. Tetanus. Lancet. 2019;393(10182):1657-68.
12. Ministry of Health. Quyết định 5642/QĐ-BYT về việc ban hành tài liệu chuyên môn "hướng dẫn chẩn đoán và điều trị một số bệnh truyền nhiễm". 2015.
13. Southeast Asia Infectious Disease Clinical Research Network. Causes and outcomes of sepsis in southeast Asia: a multinational multicentre cross-sectional study. Lancet Global Health. 2017;5(2):e157-e67.
14. Rudd KE, Johnson SC, Agesa KM, Shackelford KA, Tsoi D, Kievlan DR, et al. Global, regional, and national sepsis incidence and mortality, 1990-2017: analysis for the Global Burden of Disease Study. Lancet. 2020;395(10219):200-11.
15. Do SN, Luong CQ, Pham DT, Nguyen MH, Nguyen NT, Huynh DQ, et al. Factors relating to mortality in septic patients in Vietnamese intensive care units from a subgroup analysis of MOSAICS II study. Sci Rep. 2021;11(1):18924.
16. Dat VQ, Vu HN, Nguyen The H, Nguyen HT, Hoang LB, Vu Tien Viet D, et al. Bacterial bloodstream infections in a tertiary infectious diseases hospital in Northern Vietnam: aetiology, drug resistance, and treatment outcome. BMC Infect Dis. 2017;17(1):493.
17. Dat VQ, Huong VTL, Turner HC, Thwaites L, van Doorn HR, Nadjm B. Excess direct hospital cost of treating adult patients with ventilator associated respiratory infection (VARI) in Vietnam. PLoS One. 2018;13(10):e0206760.
18. Cifra CL, Westlund E, Eyck PT, Ward MM, Mohr NM, Katz DA. An estimate of missed pediatric sepsis in the emergency department. Diagnosis. 2021;8(2):193-8.
